# Atmospheric pressure plasma etching of Ti-6Al-4 V using SF_6_ etchant

**DOI:** 10.1186/s40712-024-00200-9

**Published:** 2025-09-11

**Authors:** Alex Bishop, Zhaorong Huang, Claudiu Giusca, Adam Bennett, Marco Castelli, Tian Long See

**Affiliations:** 1 School of Aerospace, Transport and Manufacturing, Cranfield University, Bedfordshire, MK43 0AL UK; 2 Centre of Micro/Nano Manufacturing Technology, University College Dublin, Dublin 4, Belfield, Ireland; 3 The Manufacturing Technology Centre Ltd., Coventry, CV7 9JU UK

**Keywords:** Atmospheric pressure plasma etching, Ti-6Al-4V, Surface finishing, Form correction, SF_6_ etchant, Plasma etching of metals

## Abstract

Atmospheric pressure plasma (APP) etching has been developed recently into a manufacturing technique for silicon-based materials used for large optical lenses. However, there are few reports published regarding APP etching of non-silicon-based materials. We report here the development of an APP process using SF_6_ for the etching of Ti-6Al-4 V metal alloy. Ti-6Al-4V is extensively used in aerospace and biomedical fields for its excellent properties; however, these properties also make it difficult to machine. Current techniques such as precision grinding and laser polishing can be slow, energy intensive, and cause damages and defects which reduce the lifetime of vital components. The results in this paper demonstrate effective material removal and little surface damage by APP etching of Ti-6Al-4V. Material removal rates between 0.5 and 2 mm^3^ min^−1^ were obtained, and the proposed material removal mechanism is through the formation of volatile VF_x_ and TiF_4_. These results show that APP etching is a promising technique for surface finishing of Ti-6Al-4V, particularly for large- and complex-shaped components.

**Graphical Abstract:**

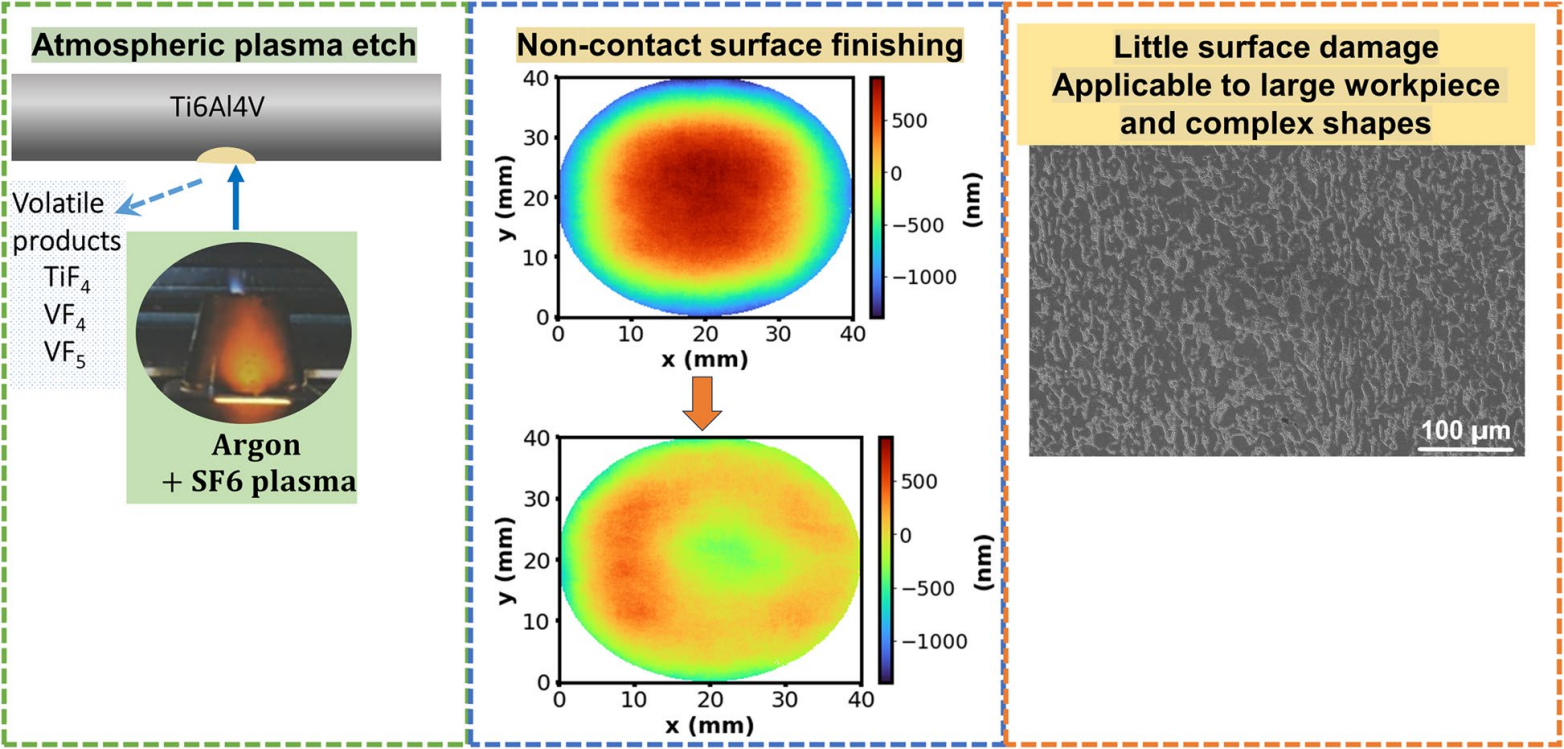

**Supplementary Information:**

The online version contains supplementary material available at 10.1186/s40712-024-00200-9.

## Introduction

Plasma etching is a commonly used noncontact material removal process for the manufacturing of precision lenses and integrated circuits due to its accuracy, selectivity, and the excellent surface finishes achievable (Leiberman & Lichtenberg 2005). While plasma etching is often done under vacuum conditions, the need for a vacuum chamber significantly increases machining time and costs for large components. Recently, atmospheric pressure plasma (APP) etching of materials has attracted extensive research interest. APP etching of silicon-based materials has been developed successfully into a key technology for the surface finishing of large precision optics (Guo et al. 2020; Krumpolec et al. 2017). However, very limited research has been published looking into APP etching of other materials besides polymers (Fricke et al. 2010) and tantalum/tungsten (Jeong et al. 1998). This work reports the development of an APP etching process capable of safely etching Ti alloy Ti-6Al-4V.

Ti-6Al-4V (Ti6Al4V) is the most common alloy of titanium, used extensively in the aerospace (Boyer 1996) and biomedical (Avila et al. 2018) sectors for its high strength at high temperatures, biocompatibility, and corrosion resistance. Due to these properties, Ti6Al4V is classed as a difficult-to-machine material (Veiga et al. 2013). The final surface finishing process of Ti6Al4V is commonly done using mechanical processes (Revankar et al. 2014) or laser polishing (Obeidi et al. 2022). However, these processes can leave subsurface damage, microfractures, and changes in the microstructure which can in turn reduce the operational life of the component (Solheid et al. 2020). Mechanical contact machining techniques used for finishing Ti6Al4V, such as precision grinding, also have drawbacks, such as high running costs, expensive tool replacement, high tool wear, long processing times (Lee et al. 2021), and substantial residual stresses (Nespor et al. 2015). Wet chemical etching (Garich & Hall 2018) such as electropolishing (Urlea & Brailovski 2017) has also been used for the surface finishing of Ti6Al4V parts; however, this can be very time-consuming and requires the use of harmful chemicals. Ion beam finishing (IBF) is a well-established technique for nanoscale alterations to a surface. While highly accurate, IBF is very slow, and the process requires a vacuum, making it unsuitable for large samples (Lee 1979).

If an APP etching process can be developed for the surface finishing for Ti6Al4V, it would leave negligible surface damage and sufficiently high material removal rate for large components. This ion energy-driven etching involves both ion bombardment and chemical etching, and the etching rate is often dominated by chemical processes (Leiberman & Lichtenberg 2005), meaning a suitable etchant must be chosen which is capable of forming volatile compounds with the target material (Flamm et al. 1983).

There are two main impacts on the plasma which need to be considered when shifting from vacuum to atmospheric pressure conditions: Firstly, the increase in ionization energy required under atmospheric pressure (Leiberman & Lichtenberg 2005), and, secondly, the reactions between the plasma and elements in the air. It has been reported that even increasing a vacuum chamber pressure from 1 to 5 Pa can lead to a significant drop in etch rates for various etchants (CCl_4_/O_2_ and CCl_4_/CCl_2_F_2_/O_2_ mixes) when etching Ti6Al4V (Blumenstock & Stephani 1989).

Numerous studies have been carried out investigating plasma etching of titanium at low pressure, using various halogen etchants (Loffler et al., 2012; Amirov et al. 2016). Blumenstock and Stephani (Blumenstock & Stephani 1989) proposed that the addition of fluorine in a chlorine plasma jet catalyzed the material removal by slowing down the decomposition of TiCl_x_ into TiO_2_. Fracassi and d’Agostino (Fracassi & d’Agostino 1992) proposed that fluorinating a titanium surface enables it to react more readily with chlorine. Li (Li et al. 2014) investigated various fluorine compounds (CF_4_, C_2_F_8_, and SF_6_) to etch BaTiO_3_ thin films in an inductively coupled plasma system. They showed using SF_6_ led to higher etch rates, and less nonvolatile by-products remained on the surface. These studies show that both fluorine and chlorine etchants can chemically etch titanium, creating volatile products, but TiCl_x_ are more volatile than TiF_x_ compounds (Norasetthekul et al. 2001). However, under atmospheric conditions, chlorine containing compounds should be avoided, and many previously used chlorine sources such as CCl_4_ or BCl_3_ have either been banned or are too dangerous to use. Chlorine radicals readily hydrolyze with water vapor in the air, which can pose a danger to the machine integrity and the operators. Metal chlorides remaining on the substrate surface will also hydrolyze, forming hydroxides and oxychlorides which can prevent etching (Coburn & Winter 1979). For this reason, the use of fluorine would be highly beneficial as compounds such as SF_6_ and CF_4_ are already widely used in industry and are deemed safe with precautions already in place.

To our knowledge, APP etching of Ti6Al4V has not been reported in literature. In this work, we try to develop an APP process using a fluorine-based etchant (SF_6_) for the etching of Ti6Al4V alloy. After obtaining initial positive feasibility results in static dwell and dynamic trench etch experiments, microstructural characterization of etched surface was combined with thermodynamic modelling to reveal the material removal mechanisms. Areal etching operational parameters for a specific sample profile were estimated based on the trench etching results. The obtained experimental results were then compared to the respective theoretically calculated ones, and the reasons for any discrepancies are discussed.

## Materials and methods

### Ti-6Al-4V alloy

Ti6Al4V is an *α* + *β* alloy, meaning it is comprised of a mixture of hexagonal close-packed (HCP) *α* and body central cubic (BCC) *β*-crystal structures. In general, the HCP phase provides strength, and the BCC phase provides ductility. In Ti6Al4V, aluminum is the *α*-phase stabilizer, and vanadium is the *β-*stabilizer. Depending on the quantity of each, the *β*-transition temperature (*β*-transus) can range from ∼700 °C to over 1000 °C (Lutjering & Williams 2007). Ti6Al4V contain between 5 and 30% volume of *β*-phase (Boccchetta et al., 2021).

Ti6Al4V samples were purchased from TIMET UK Ltd., measuring 50 × 50 × 16 mm^3^. The material composition was given by the supplier as 5.5–6.75% wt. aluminum, 3.5–4.5% wt. vanadium, and up to 1.145% wt. other impurities, with the remainder composed of titanium.

The as-received samples were sanded, polished, and cleaned to allow for interferometer measurements before and after etching. Sanding was carried out by hand using alumina (40–180 grit) and silicon carbide (240–2400 grit) sandpapers to initially smooth the surface. Hand sanding was preferred to prevent local crystal structure changes, thermal damage, or residual stresses which may be induced through grinding (Malkin & Guo 2007). The polishing slurry used was L1 finishing liquid from LAM PLAN®, specifically designed for polishing of Ti alloys. The cleaning process involved an initial wash with de-ionized water and then isopropanol (IPA) and dried with clean dry air.

### Atmospheric pressure plasma system

Plasma etching was carried out using the Helios-1200 machine (RAPT Industries Inc., USA). The energy coupling in the plasma torch can be either electrically dominated (E mode) or magnetically dominated (H mode) due to the design of the copper coil (Fig. S1). At low powers, the torch operates in E mode, and at high powers (above ∼800W), there is a transition to H mode, leading to an increase in plasma temperature and ionization (Yu 2016; Castelli 2012). Figure S1a shows a cross section of the torch, and the coil has five helical turns, the top 3 of which are water cooled with the bottom 2 made from solid copper. The purpose of this design is to allow for plasma ignition without the need for an additional ignition device such as a Tesla coil. The processing was done using an argon plasma at flow rates of 20 L min^−1^ as a shield gas and 0.8 L min^−1^ Ar (90%)/SF_6_ (10%) mix as the reactive species. The pure argon is required to act as a curtain around the reactive species, preventing reactions with the air. The operating power was 1.2 kW at a frequency of 40.25 MHz, and the stand-off (top of the torch to sample surface) distance was 6 mm, which previous experiments showed to be the distance where the greatest concentration of fluorine radicals was present (Castelli 2012). Table S1 summarizes these operational parameters used in the experiments. At these operating conditions, the torch operates in H mode and can be considered thermal, where the electron temperature is approximately the same as the heavy particle (ions/neutrals) temperature.

Most of the operating conditions of the plasma etching experiments throughout the results presented in this work were kept constant, with the dwell time, raster pattern, and plasma beam moving speed (actually, sample moving speed) being the only variables. Other operating parameters such as the stand-off distance, input power, pitch size, and reactive gas composition/concentration have been previously optimized for the etching of Si-based materials (Castelli 2012). This work builds on previous work by applying similar principles to an entirely new substrate, i.e., metals, and focusing on the plasma-surface interaction. Initial tests showed that the material removal is negligible with pure argon plasma (Fig. S2). This confirms the material removal contribution from ion sputtering is negligible.

### Theoretical description of plasma etching

The etching rate of a reactive plasma jet is dependent on the chemical reaction rate, which in turn is temperature dependent. In order to model the plasma etching process, the material removal rate must be determined and the relevant parameters be identified. The material removal rate function of a plasma etching process, R(x, y), can be estimated as the convolution of the beam function, B(x, y), and the dwell time function τ(x, y) as shown in Eq. (1) where * signifies the convolution operator (Drueding et al. 1995) and *x* and *y* are coordinates of the plasma beam. This is based on convolution algorithm used for ion beam figuring. Here, the dwell time function τ(x, y) has units of s mm^−2^ and describes the amount of time the torch dwells over a given area. The beam function is taken as the characteristic footprint of a given plasma torch and workpiece, typically a 3D Gaussian, and has units of nm s^−1^. These functions are determined via multiple experiments to achieve a trend of etch depth against time (Drueding et al. 1995).

1 $$\begin{aligned} R\left(x,y\right)= \int\nolimits_{-x}^{x}\int\nolimits_{-y}^{y}B\left(x-u, y-v\right)\bullet \tau \left(u,v\right)dudv=B\left(x,y\right)\\ \star \tau \left(x,y\right) \end{aligned}$$

For areal etching, a technique of dividing an area into strips is adopted where each strip has a width and a velocity function associated with it (Drueding et al., 1995). The so-called partial Cartesian discretization of the Eq. 1 is approximated as follows in Eq. (2).

2 $$R\left(x,y\right)= \sum_{j} \int\nolimits_{-\infty }^{x}B\left(x-u, y-v\right)T\left(u,{v}_{j}\right)du\Delta v$$

The simplified dwell time function T(u,v) is determined by the beam moving speed V(u) and the strip width Δv, shown as in Eq. (3) (Drueding et al. 1995).

3 $$T(u,v)\cong \frac{1}{V(u)\Delta v}$$

So, if the beam moving speed and the strip width are defined, and the beam function B(x, y) is known, the material removal function R(x, y) are determined.

The beam function B(x, y) is usually defined as a symmetrical 3D Gaussian, shown in Eq. (4), with A = 143 nm s^−1^, based on the derivative of a linear relationship for etch depth against processing speed, and *σ* was calculated as $FWHM/\left(2\sqrt{2\text{ln}\left(2\right)}\right)$, where FWHM is the full width at half maximum as determined from the trench etching. The average FWHM observed during the trench experiments was measured as ~ 10 mm.

4 $$B(x,y)=A\bullet \text{exp}\left(\frac{{x}^{2}+{y}^{2}}{2{\sigma }^{2}}\right)$$

### Plasma etching procedures

Initial experimental stationary dwells were carried out at the center of the Ti6Al4V surfaces to determine time dependency of the material removal rate. Building on this, trenches were then etched to show temporal and spatial stability of the etching process. The beam moving speed for the trenches was varied from 50 to 250 mm min^−1^ in 50 mm min^−1^ intervals. These experiments showed the dynamic etching requires the surface to be preheated to etch a clean trench. The preheating pass used a pure argon plasma jet at an input power of 1.2 kW and 6- mm standoff at a speed of 50 mm min^−1^. This was kept consistent throughout the experiments. This pass was carried out directly before the SF_6_ was injected into the plasma stream.

Initial areal etching experiments were carried out by overlapping trenches at a constant speed at 100 mm min^−1^ and at a pitch width (separation of strips Δv) of 5 mm. The pitch of 5 mm was chosen as half the average FWHM determined from the trench etching. This rastering back and forth at a constant speed is called a neutral removal process (Fig. S3a). This was first done by overlapping three trenches (to observe if the pitch of 5 mm would result in a smooth removal footprint) and then seven, to cover the entire measured surface. An alternate pass pattern, as shown in Fig. S3b, was also employed, to investigate any effect of the raster pattern on the etching rate.

**Fig. 1:**
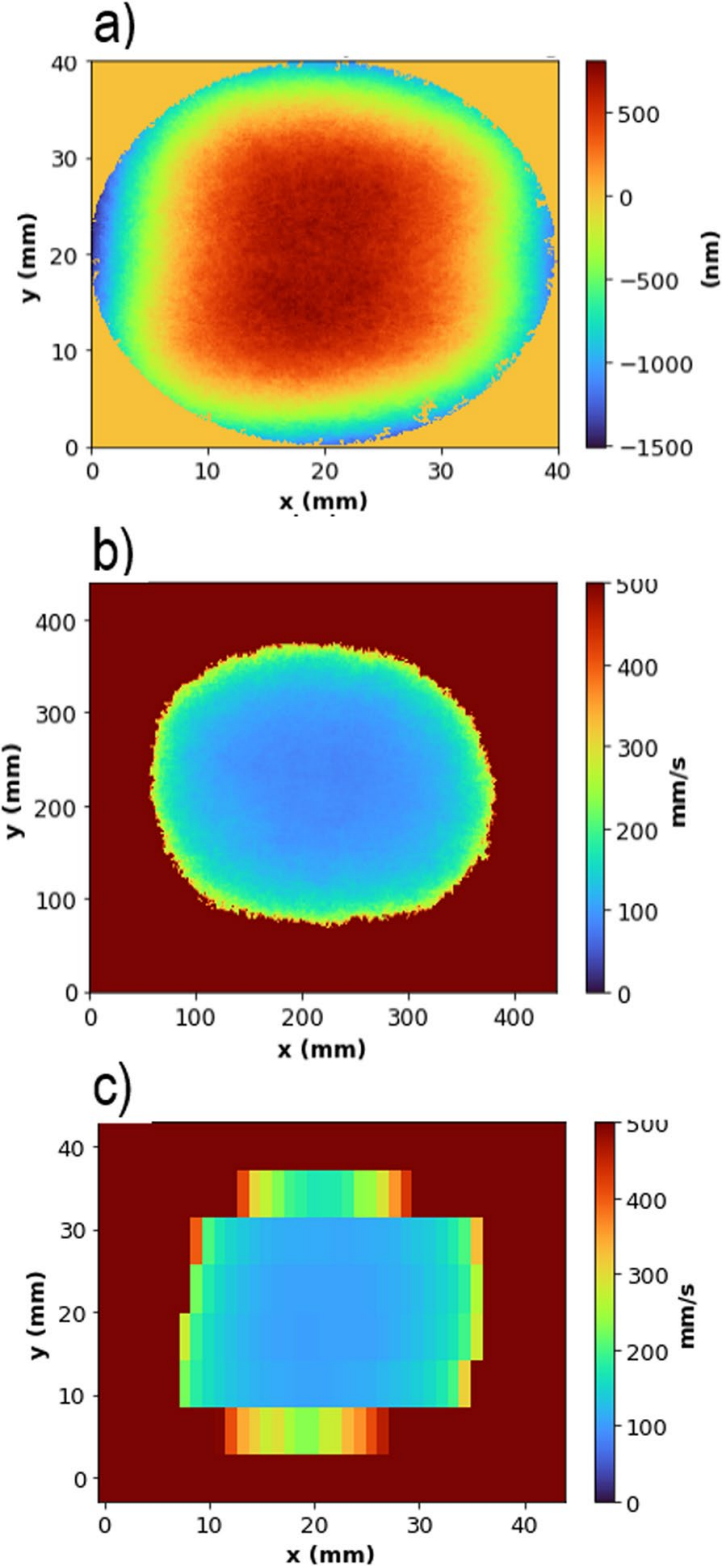
Steps taken to build the velocity map V (x, y) used for areal plasma etching: **a** The required material removal (etch depth) pattern. **b** Each pixel assigned a processing speed V (calculated from $\text{etch depth}=\text{334,450}\bullet {\text{V}}^{-1.36})$ to reduce the surface height to 0 and (**c**) averaged velocity map for areas of 5 mm × 1 mm

For simplicity, the required velocity function V(u) was obtained through an empirical method, as shown in Fig. 1. For a particular etching requirement (Fig. 1a), the relationship between the etching depth and speed V was obtained from the trench experiments, i.e., $\text{etch depth}=334450\bullet {\text{V}}^{-1.36}$. The processing speed V was set to result in a height of 0 nm for each pixel. Beam moving speed V was calculated for each pixel in the surface measurement matrix, leading to a velocity map (Fig. 1b). This velocity map was averaged into areas of 5 mm × 1 mm to input into the Helios-1200 machine (Fig. 1c), which does not have enough memory or the capability to change velocity at each pixel (∼193,000 pixels in each measurement area). The velocity was set to 500 mm min^−1^ when the height of the pixel was equal or below 0, i.e., when no etching was required.

Using the above obtained beam function and the dwell time function (obtained from Eq. 3), a material removal function R(x, y) can be calculated. This calculated material removal pattern was compared to the actual obtained experimental pattern. As the time function was the same for the experiment and the calculation, this comparison can be used as an investigation to the adopted beam function, or, precisely, the assumptions used in obtaining the beam function, which are discussed in the “ Areal plasma etching” section.

### Methods for surface characterization

The material removal was characterized using FST10 (Fisba Optik, Switzerland) and DynaFiz® (Zygo, USA) interferometers in phase-shifting mode. The surface of each sample was measured before and after plasma processing to determine the amount of material removed. Each measurement was carried out using a Φ40-mm measurement mask, resulting in a 1020 × 1020 pixel surface using the FST10 interferometer and a 440 × 440 pixel surface using the DynaFiz®. The surface roughness was measured using a Form Talysurf 120-L profilometer (Taylor Hobson, UK). The areal surface roughness was characterized using a Talysurf 6000 (Taylor Hobson, UK) coherence correlation interferometer (CCI).

Scanning electron microscopy (SEM) and energy-dispersive X-ray spectroscopy (EDS) analysis were carried out using a TESCAN Vega-3 SEM equipped with AZtec from Oxford Instruments for EDS. Results were obtained at three stages of the process: (i) before etching, (ii) after etching, and (iii) after etching and polishing. The samples required post-etching polishing for a few seconds since an opaque layer was formed which prevented optical measurements using the DynaFiz interferometer. SEM and EDS results were obtained at step (ii) to characterize the nature of this opaque layer. 3D microstructural characterization of the etched and polished surface was carried out using a Bruker Veeco Dimension 3100 atomic force microscopy (AFM) system in tapping mode and a measurement area of 25 × 25 µm^2^.

## Results and discussion

### Thermodynamic simulations

**Fig. 2:**
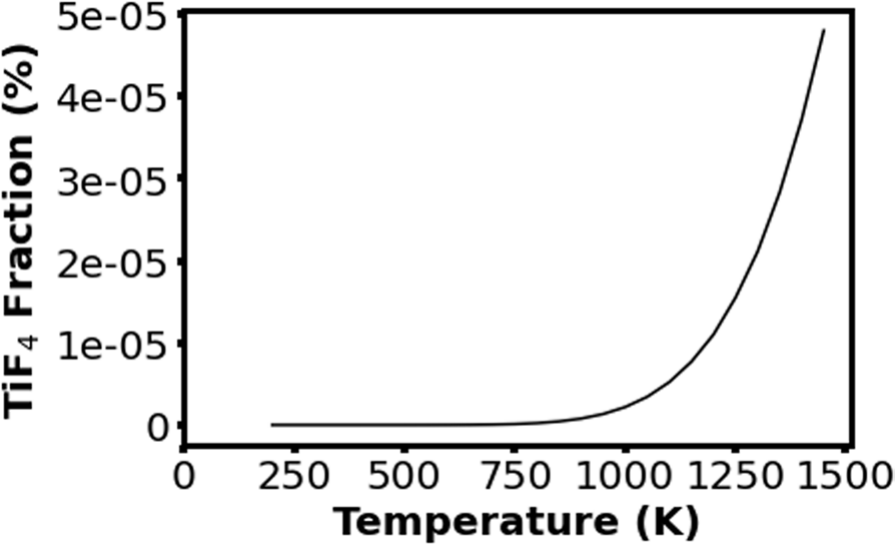
Fraction of TiF_4_ formed at temperatures between 500 and 1500 K as calculated from FactSage™. The amount of TiF_4_ increases exponentially with temperature

Thermodynamic simulations at equilibrium were carried out using FactSage™ software, which calculates the products formed under equilibrium conditions given the reactants and their quantities. This software calculates the products based on the minimization of Gibbs free energy. The surface temperature experienced by the substrate during etching when using the Helios 1200 torch is estimated to be ∼850 K based on previous work (Revankar et al. 2014). From this, simulations were run with input reactants as Ti, Al, V, and SF_6_ in their respective quantities (Table S2). Table 1 shows the top products formed at equilibrium at 850 K. The melting and boiling points for each compound are also listed in Table 1. Fractions of VF_**x**_ compounds were not available since the software predicts negligible amounts formed due to the low concentration of vanadium; however, the melting and boiling points for VF_4_ and VF_5_ are also included in Table 1. These thermodynamic simulations were carried out at temperatures between 500 and 1500 K in 50-K intervals. Figure 2 shows the calculated fraction (%) of TiF_4_ produced follows an exponential trend as temperature increases. If TiF_4_ is the only volatile titanium product formed, these simulations suggest that the etch rate is exponentially related to the surface temperature.

**Table 1:** Thermodynamic simulation results from FactSage™ showing the most formed products when Ti, Al, V, and SF_6_ coexist at 850 K and their respective melting and boiling points

Product	Fraction (%)	Melting point (°C)	Boiling point (°C)
TiF_3_	80.9	1200	1400
TiF_2_	15.2	^a^	^a^
AlF	3.8 × 10^−2^	^a^	^a^
AlF_3_	6.5 × 10^−4^	Sublimates	1297
AlF_2_	4.2 × 10^−4^	^a^	^a^
TiF	5.6 × 10^−7^	^a^	^a^
TiF_4_	4.3 × 10^−7^	Sublimates	284
VF_4_	-	Sublimates	325
VF_5_	-	20	48

^a^Melting and boiling points not available in literature

There are several limitations of these simulations. The main problems are as follows: (i) it does not take account of the ionization of the plasma jet, and (ii) it assumes equilibrium conditions. Both are not valid for the plasma etching in this work. However, these simulations at least can provide a list of possible products. As ionization of the gases in the plasma jet can increase the reaction activities substantially, the formation of some products could be much more than the predicted by this software. The fact that the addition of SF_6_ to the plasma jet can etch Ti6Al4V alloy, as will be shown below, suggests that much more TiF_4_ may have been formed than the predicted by this simulation.

### Stationary and dynamic plasma etching

**Fig. 3:**
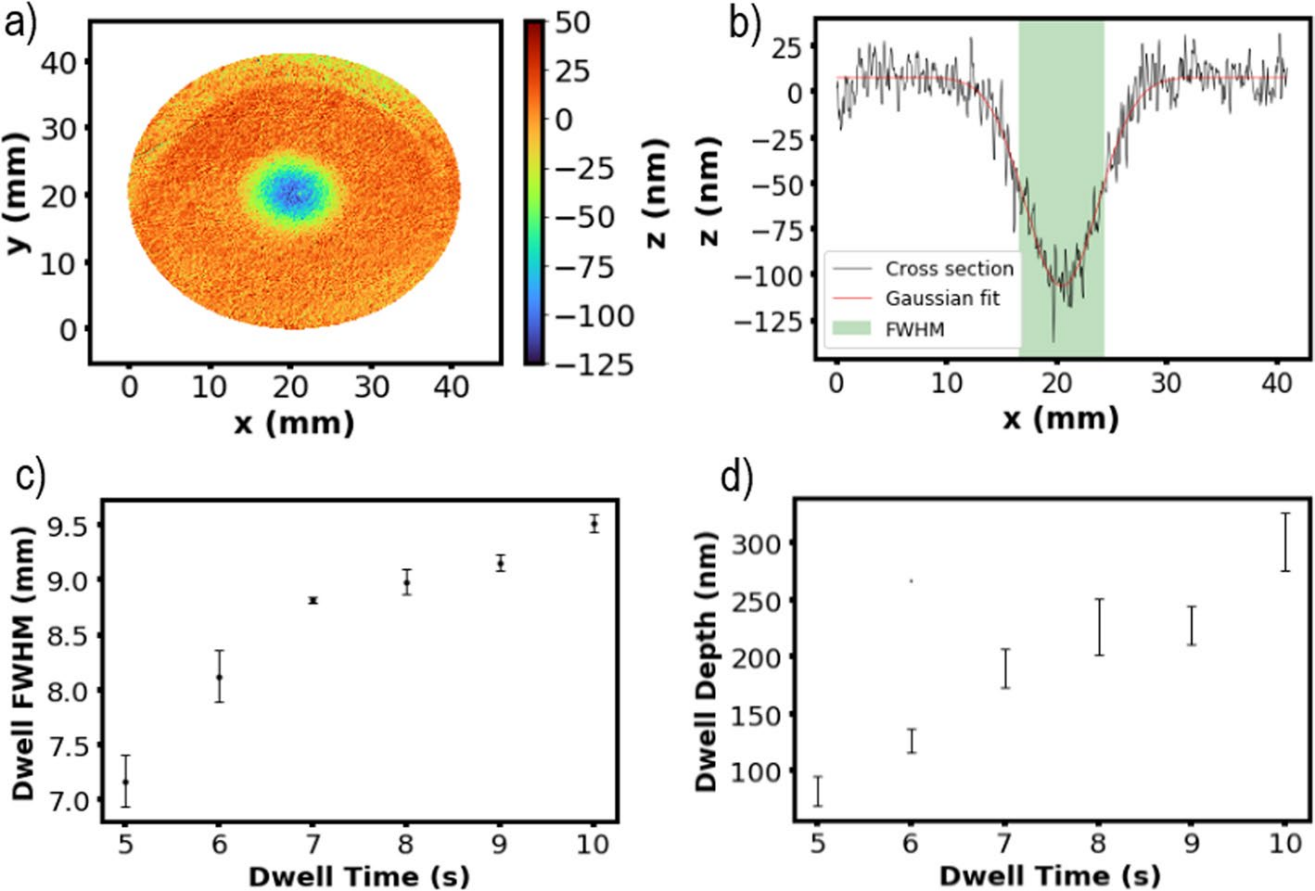
Static dwell experimental results. **a** 2D plot of an interferometer measurement of the pitch footprint after a 5 s dwell. **b** Its 2D footprint profile in *x* direction. The profile fits very well by a Gaussian function. **c** Dwell full width at half maximum (FWHM) and (**d**) dwell depth against time between 5 and 10 s dwells. Error bars show ± standard deviation of three results at each data point

Despite the unpromising simulation results, initial experimental test results proved more encouraging. Preliminary plasma etching tests for a few seconds turned the surface optically opaque, and no optical measurements could be carried out (Fig. S4a). However, after hand polishing for a few seconds, these samples were reflective enough for optical measurements (Fig. S4b). Figure 3a shows an example of a dwell footprint as measured from the FST10 phase-shifting interferometer after a 5 s dwell time and a profile along the *x*-axis in the center of the sample (Fig. 3b). The profile confirms that the etching footprint resembles a Gaussian function. Figure 3c and d shows the variation in FWHM and etch depth against dwell time respectively. These results were taken from 3 repeats at each dwell time with the error bars showing ± standard deviation of the results. Dwell times below 5 s showed no measurable etching. The increase in FWHM with dwell time up to 10 s suggests the torch footprint is not stable below 10 s. Even after a dwell time of 10 s on Ti6Al4V, the FWHM has only reached ~ 9.5 mm and does not appear to be levelling off. This is very different from the APP etching of Si-based materials where a plateau in FWHM ~ 11 mm is reached after a dwell time of 1 s (Solheid et al. 2020).

Initially trenches showed unstable etching as shown in Fig. S5a. To solve this, the samples were preheated by passing a pure argon plasma over the surface immediately before etching at a speed of 50 mm min^−1^. This speed was chosen to sufficiently heat the surface, and measurements showed this preheating pass did not remove any additional material. This preheating led to clean trenches as shown in Fig. S5b, as well as higher etch rates than expected based on the stationary dwells (during the time for the torch to travel the length of FWHM, i.e., 10 mm) as the latter had no preheating. Fig. S6 shows the trench footprints as measured by phase-shifting interferometer techniques for processing speeds between 50 and 250 mm min^−1^.

**Fig. 4:**
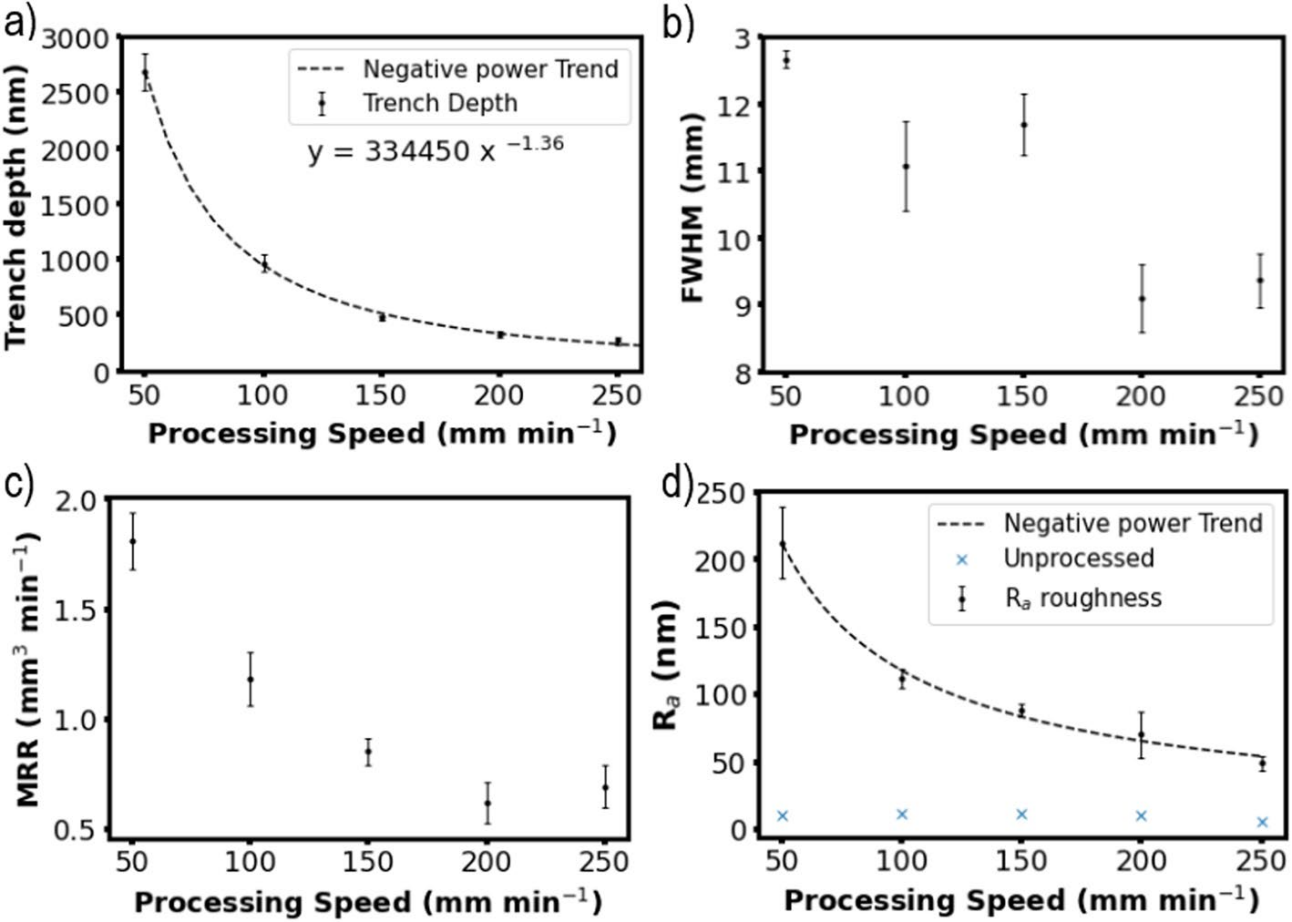
Atmospheric pressure plasma dynamic trench etch experimental results. **a** Maximum trench depth, (**b**) full width at half maximum (FWHM), (**c**) materials removal rate (MRR), and (**d**) surface roughness R_a_ against beam processing speed for trenches between 50 and 250 mm min^−1^ after preheating with an argon plasma. The trend shown in (**a**) was calculated as 334,450 · x^−1.36^. The trend line equation in (**d**) is calculated as 5841x^−0.85^. The error bars show ± standard deviation of three separate results at each data point

The etch depth varied against beam moving speed in an inverse power way between 50 and 250 mm min^−1^ after doing the preheating pass (Fig. 4a). The trend shown is of the form $a\bullet {x}^{-b}$ where *a* = 334,450 and *b* = 1.36. Again, the error bars show ± standard deviation of three separate results at each data point. Figure 4b shows the FWHM of the torch footprint is variable with respect to beam speed. This implies that the surface-torch interface has not reached a thermal equilibrium since the FWHM has not reached a plateau. With a variation in FWHM from ∼9 to ∼12.5 mm, 10 mm was taken as an average FWHM. From these etching footprints, the material removal rate (MRR) can be calculated (Eqs. S2 & S3 in the Support Information). Figure 4c shows how MRR varies with beam speed. Comparing this with typical MRR for alternative techniques, this plasma etching process leads to MRR values (0.5 mm^3^ min^−1^–2 mm^3^ min^−1^) at the lower end of typical APP etching MRR of silicon-based materials (0.1 mm^3^ min^−1^–50 mm^3^ min^−1^). However, this is still significantly greater than alternative techniques such as IBF (∼0.01 mm^3^ min^−1^) and comparable to that of magnetorheological finishing (MRF) (0.3 mm^3^ min^−1^–13 mm^3^ min^−1^) for Si-based materials [29]. It is also worth noting the repeatability of the process. The error bars shown in Figs. 3d and 4a are narrow (< ± 40 nm in all except the trench depth at 50 mm min^−1^) over multiple samples. This shows the plasma etching process is highly deterministic and repeatable when etching an individual trench or dwell within the time period investigated.

The roughness of the etched surface *R*_a_ was measured using profilometry across the trenches. This was found to increase with the decreasing raster speed (Fig. 4d). Before plasma etching, the *R*_a_ was measured to be between 6 and 12 nm. This was increased to 50 nm after etching at 250 mm min^−1^ and reached more than 200 nm at 50 mm min^−1^.

### Microstructural and compositional analysis of the surfaces

**Fig. 5:**
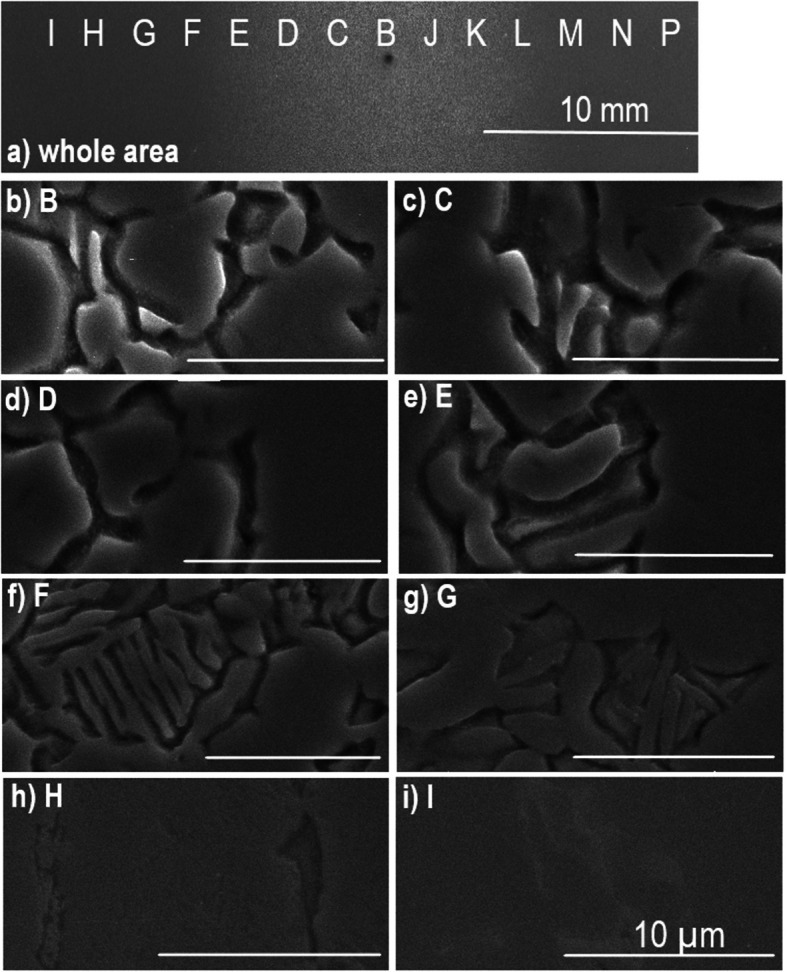
SEM images of Ti6Al4V surface after plasma etching and polishing. **a** Wide-field image at low magnification. A trench etched at a beam moving speed of 100 mm min^−1^ can be seen in the center of the image. **b**, **c**, **d**, **e**, **f**, **g**, **h**, **i** Are images taken at points B–I shown on (**a**), respectively. Each image is taken 2 mm apart

SEM images were taken for all the three stages: before etching, after etching, and after etching and polishing. Figure 5a shows a low magnification secondary electron (SE) SEM image of a Ti6Al4V surface after plasma etching and polishing. The trench etched can be seen in the center of the image (bright contrasted) which was carried out at a beam moving speed of 100 mm min^−1^. SEM images were taken at various points across the width of the trench to determine variations in surface morphology and grain structure across the area of plasma influence. These points are labelled in Fig. 5 as B–P. The distance between two adjacent measurement points, such as from B to C, is 2 mm. In total, 14 images were taken over a width of 26 mm across the direction of etching. The center of the trench appears to be between B and C in Fig. 5a. The etching footprint as shown in Fig. S6b suggests that the etching is symmetric; indeed, similar morphology was observed for the pairs of B and C, D and J, E and K, F and L, G and M, and H and N (where the morphology was very much the same as that for the untreated sample). Figure 5b, c, d, e, f, g, h and i shows SEM images taken at the positions shown in Fig. 5a at B–I, respectively, across half of the trench. These images show that the etching is the deepest at the centers B (b) and C (c) and progressively reduces towards the edge G (g) and H (h). At I, the surface was almost featureless (i), the same as for the untreated area.

**Fig. 6:**
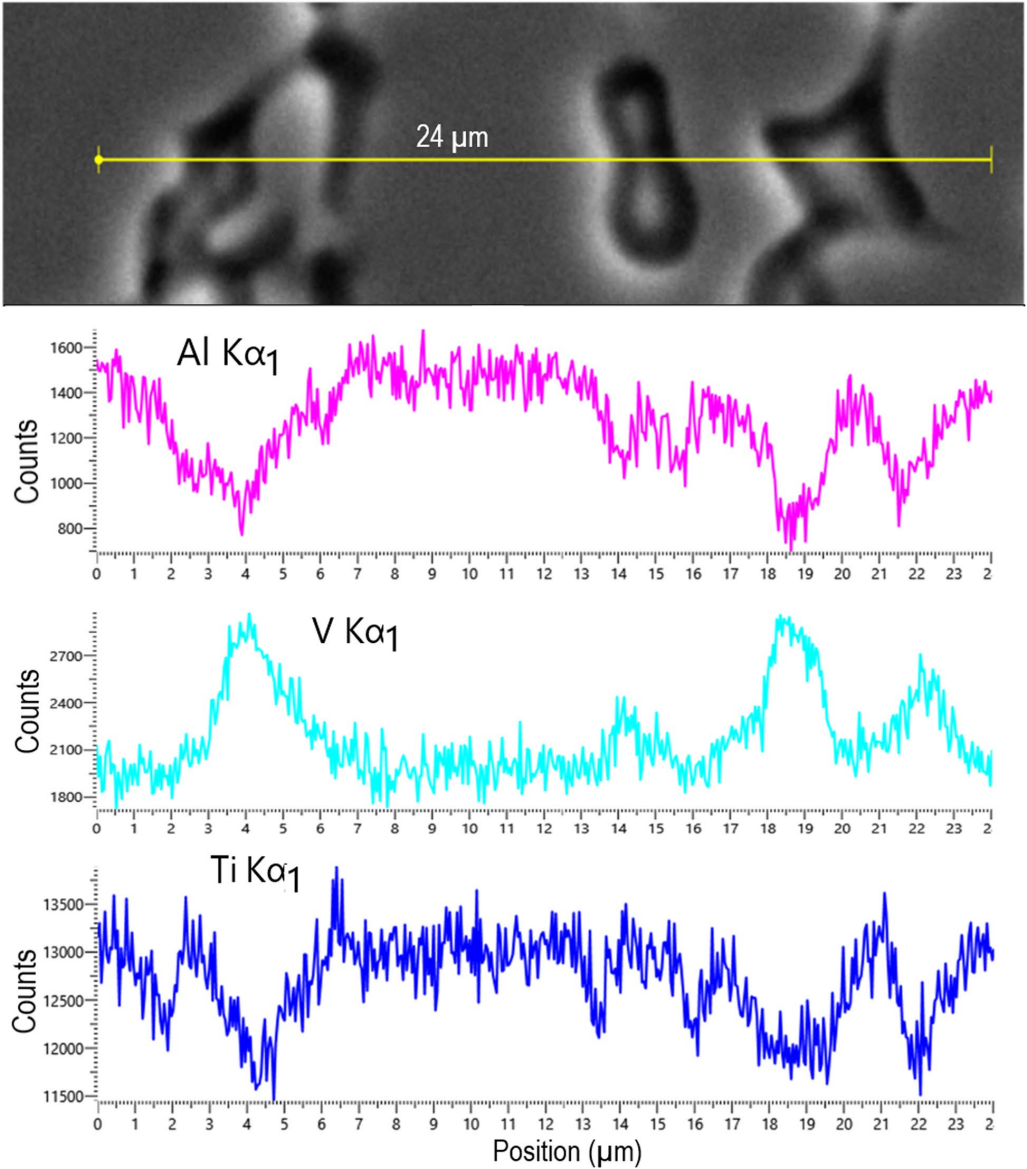
SEM image and EDS line scans showing chemical composition of aluminum, vanadium, and titanium (from top to bottom respectively) across grain boundaries along the line shown in the SEM image at the top. EDS line scan results indicate that the dark contrasted regions are vanadium-rich *β*-phase and the bright contrasted grains are aluminum-rich* α*-phase

**Fig. 7:**
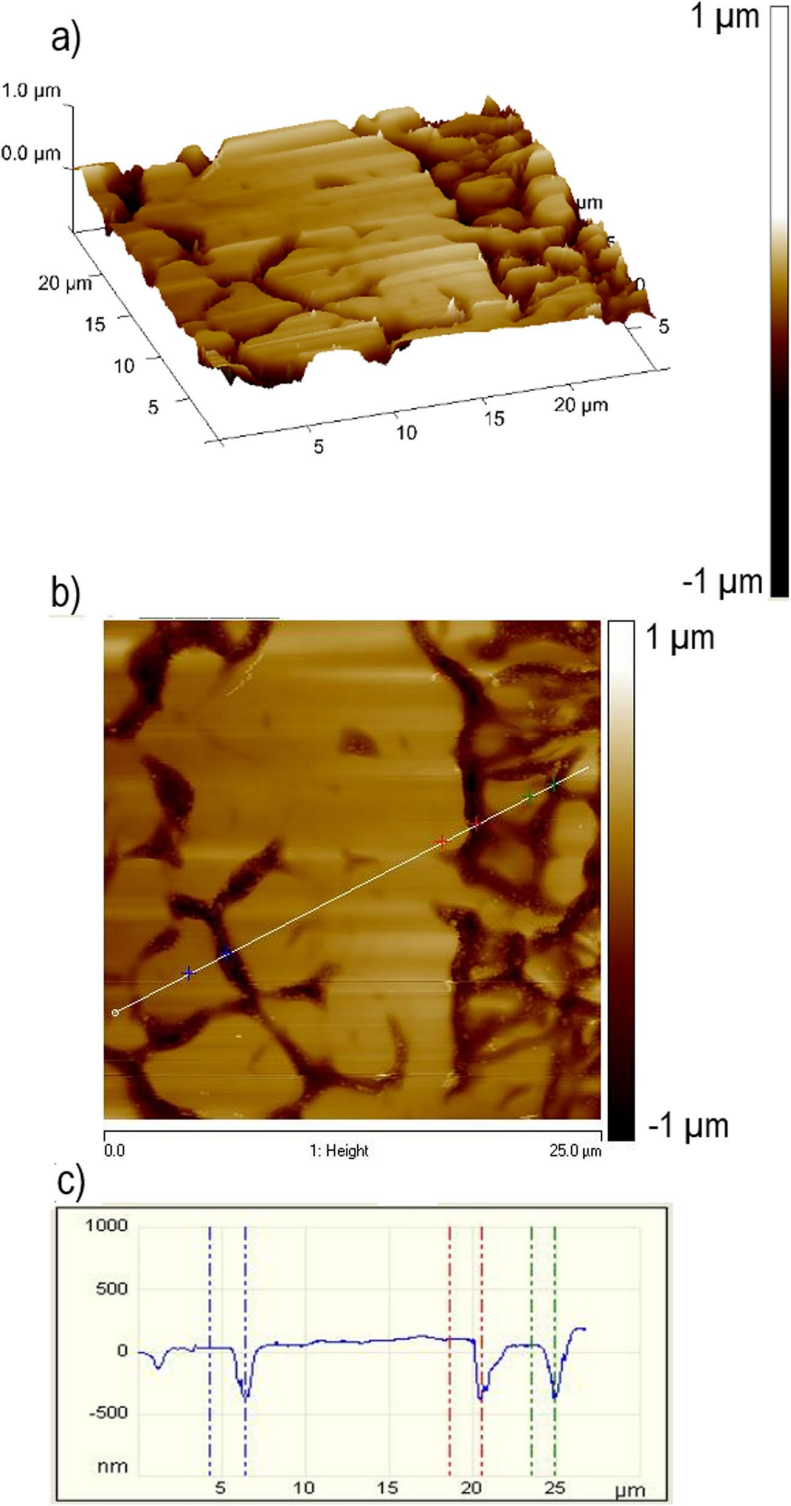
Atomic force microscopy scan results. **a** 3D and (**b**) 2D scan taken at the center of a trench etched at 50 mm min^−1^ after etching and polishing. **c** Shows the profile across the line in (**b**). The results indicate that the *β*-phase grains are preferentially etched than the *α*-grains

**Fig. 8:**
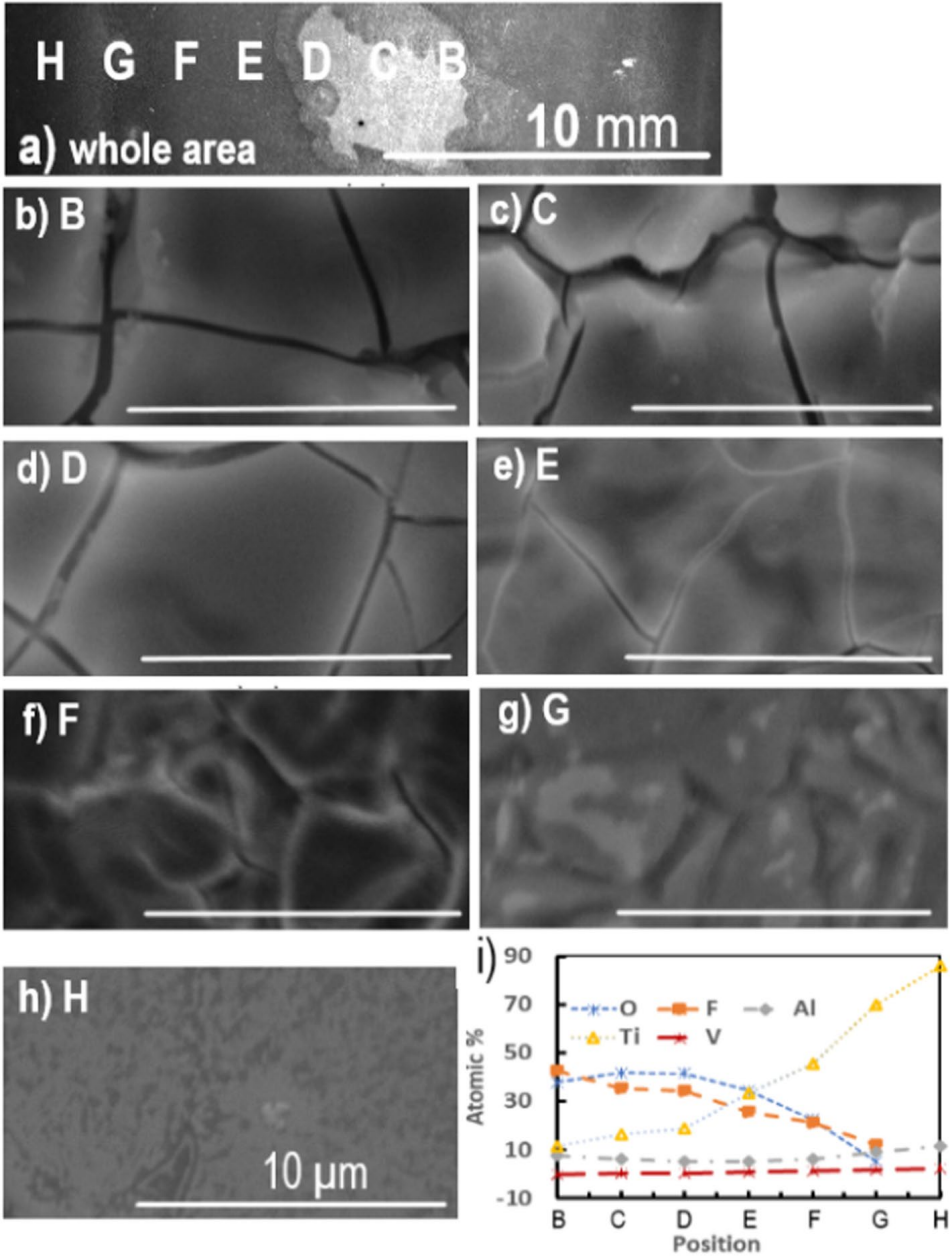
SEM images and surface compositions of Ti6Al4V surface immediately after plasma etching at 50 mm min^−1^. **a** At low magnification. **b**, **c**, **d**, **e**, **f**, **g**, **h** Are secondary electron images taken at points B–H shown on (**a**), and the scale bar is 10 µm. Each image is taken 2 mm apart. **h** Is the image from the untreated area. **i** Atomic percentage (%) of different elements across the width of the plasma footprint for positions B–H in (**a**) immediately after plasma etching

Figure 6 shows a back-scattered electron (BSE) SEM image and EDS line-scan results across grain boundaries with the line profiles for Al, V, and Ti peaks. It shows clearly that the dark region is vanadium rich and poor in titanium and aluminum. The bright contrasted region is relatively aluminum rich. These suggest that the dark region is the *β*-phase, and the bright contrasted region is the *α*-phase (Fig. 6 top & Fig. 7). The apparent Ti reduction is probably due to the existence of small amount of etch residue in the *β*-region which has a much lower Ti concentration (this will be discussed below in Fig. 8). If so, the morphology of the sample as shown in the above SEM images corresponds to an equiaxed structure as opposed to lamellar or bimodal structures (Lutjering & Williams 2007; Bocchetta et al. 2021). The α:β ratio for the samples used here was then determined, using thresholding in ImageJ, to vary between 8.2 and 19.5% *β* for the SEM images in Fig. 5b, c, d, e, f, g and h, which sits within the range between 5 and 30% of the *β*-phase stated in literature. The average ratio was determined as 0.87α:0.13β. This is similar to the α:β ratio of 0.83α:0.17β determined from the SEM images of the un-etched areas. An example of a thresholding SEM image is shown in Fig. S7 for point B (in Fig. 5a), where Fig. S7a shows only the *α*-phase and Fig. S7b shows only the *β*-phase. The original SE SEM image, Fig. S7c, is included as a reference. The same equiaxed morphology and similar *α*-*β* ratios between the clean and etched surfaces confirm that the plasma etching process has not exceeded the *β*-transus temperature of 1268 K (Lutjering & Williams 2007).

The above SEM images suggest the *β*-grains were etched more than the *α*-grains. AFM measurements were then carried out to determine the extent of this preferential etching. The scan shown in Fig. 7a was carried out at the center of a trench after plasma etching and polishing. The trench was etched at a speed of 50 mm min^−1^. The results clearly show the *β*-phase was etched significantly more than the surrounding *α*-phase. Line scans confirm that the *β*-phase has been etched up to 500 nm deeper than the surrounding *α*-phase, where the blue, red, and green points are taken to compare the relative height of the surface (Fig. 7b & c). The narrow width of the *β*-grains also means that phase-shifting interferometer (PSI) measurements will not be able to detect the *β*-phase, and only the *α*-phase is detected. As the trench depth is ~ 1 µm, this leads to the conclusion that the *β*-phase is etched up to 50% faster than the *α*-phase. This preferential etching of the *β*-phase means that the ratio of *α*-*β* phases and the grain size of each phase for a particular Ti6Al4V sample will affect the etch rate and surface topology after etching. It is noteworthy to compare this to the electrochemical dissolution behavior of Ti6Al4V. Experiments investigating the corrosion resistance of Ti6Al4V have shown that in a 15% wt. NaCl solution, the *α*-phase is preferentially etched, compared to the preferential etching of the *β-*phase seen in the reactive plasma here using SF_6_ (Li et al. 2022).

### Mechanism of the plasma etching of Ti6Al4V

Microstructural characterization was also carried out for the sample surface immediately after the plasma etching procedure, before any after-processing polishing was done. Figure 8a shows a low magnification SE SEM image of a Ti6Al4V surface immediately after an APP etching at a beam speed of 50 mm min^−1^. As above, SEM images were taken at various points across the width of the trench to determine variations in surface morphology and grain structure across the area of plasma influence. These points are labelled in Fig. 8a as B–H and span 12 mm with each point 2 mm apart. The corresponding SEM images for each point are shown in Fig. 8b, c, d, e, f, g and h.

At first glance, the surface appears cracked towards the center of the trench, as seen in Fig. 8b and c. However, upon further investigation and comparing the morphology to the other SEM images, it can be deduced that this is the residue or redeposition from the plasma etching process. This residue is thick enough at the center of the trench (Fig. 8b, c, d) that the secondary electrons cannot penetrate the redeposited layers to reach the detector, so only the redeposited material surface can be seen. However, away from the center of the etched trench, such as in Fig. 8e, the shape of the etched surface (dark contrasted areas) can be seen together with the redeposited material, indicating the surface shape of the redeposited materials follows the morphology of the preferentially etched *β*-grains. Further away from the center, at F and particularly at G (Fig. 8g), the shapes of the etched *β*-grains (dark contrasted) become more apparent, and the redeposited materials are less obvious but still visible. No cracks of the redeposited layer can be found in G (Fig. 8g). Further away from the center of the trench, such as at position H, there is little redeposition (Fig. 8h), and the slightly etched *β*-grains are observable. Outside of the trench footprint, the almost featureless morphology resembles that of the clean, un-etched surface.

EDS was then employed to analyze the chemical composition of the residue material. Figure 8i displays how the chemical composition varies with respect to the position across the plasma pitch. It illustrates how the amounts of fluorine, oxygen, and titanium correspond to the amount of etching taking place. At position H in Fig. 8a (outer part of the plasma footprint), only titanium, aluminum, and vanadium are present with no oxygen or fluorine present. Towards the center of the trench, at position B, the atomic percentages are as follows: *O* = 37.9%, *F* = 42.7%, *Al* = 7.9%, *V* = 0.1%, and *T*i = 11.4%. This means the amount of Ti is reduced significantly, with Ti/Al (atomic) ratio reduced from 7.4 at position H to 1.4 at position B. The concentrations of oxygen and fluorine, however, increase significantly towards the center of the trench, reaching a combined concentration of over 70% at positions B, C, and D. Vanadium is reduced towards the trench center to ∼0.2% at positions B, C, and D. The concentration of aluminum remains relatively stable, varying between 5.5 and 7.9% from positions F to B. The very brief (a few seconds) cleaning and polishing process after the plasma etching returns the ratio of elements to approximately the pre-etching composition, as aforementioned. This proves that this presence of oxygen and fluorine is in fact residue which is only adsorbed onto the surface in the form of fine dusts, and not chemically bound to the surface.

These results are summarized as below:

(i) Vanadium does not exist in the redeposited materials, meaning all the etched vanadium is evaporated as volatile compounds.
(ii) The titanium concentration in the redeposited materials is less than 20 at. %, much less than the ~ 90 at % concentration in the original Ti6Al4V, and the Ti/Al ratio is reduced significantly from its original value, implying much of the titanium is also evaporated during etching as volatile compounds.
(iii) The redeposition has a rough composition of between 20 and 30% at. (Al, Ti):80–70% at. (O, F).
(iv) The redeposition is concentrated within the plasma footprint. Outside of the plasma jet area of influence, the elemental composition and morphology are close to that of a clean, un-etched sample (~ 8).

By combining the above SEM and EDS results with the FactSage simulations, the plasma etching mechanism can be proposed. Firstly, it requires time to heat the sample surface close to the plasma temperature, which is ∼850 K. At this temperature, some metal fluoride compounds, such as TiF_4_ (b.p. = 557 K), VF_5_ (b.p. = 321 K), and VF_4_ (b.p. = 598 K), are volatile; hence, the vanadium will be chemically etched and removed in the form of VF_4_ and VF_5_. The facts that (i) TiF_4_ is the only volatile titanium fluorine compound around 850 K, (ii) Ti6Al4V can be etched at relatively high MRR by SiF_6_ containing plasma, and (iii) the FactSage simulation result that a small amount of TiF_4_ and a large amount of TiF_3_ are formed at this temperature under equilibrium conditions prompt us to suggest that the main process of material removal is the formation of TiF_4_ volatile compound in the plasma. This is most likely through the formation of TiF_3_ (or TiF_2_ to a less extent) first and then reaction with the fluorine radicals to form TiF_4_. This is because TiF_3_ and TiF_2_ are thermodynamically favorable to be formed at around 850 K, but this temperature is much less than the boiling temperatures of TiF_3_ (higher than 1600 K) or TiF_2_ (the boiling point of TiF_2_ is unknown but likely to be higher than that of TiF_3_); hence, Ti is unlikely to be lost as TiF_3_ or TiF_2_. However, we could not rule out the direct formation of TiF_4_ in the plasma environment. As for the aluminum removal, we suggest this is a consequence of the titanium and vanadium removal. This is based on the facts that (i) there should be no volatile aluminum containing compound at this temperature, (ii) less than 7% wt of aluminum exists in the Ti6Al4V alloy, and (iii) the concentration ratio (atomic) of Ti/Al/titanium is close to 1.4 at the center of the etching residue, about 1/6 of the nominal concentration ratio (~ 8) of the clean Ti6Al4V. This suggests a significant amount of aluminum, if not all, remained in the residue or the redeposition. Based on this mechanism, it can be explained that the lack of etching seen in dwell times below 5 s, and in the trench without preheating, is due to the time required to heat the sample to sufficient temperature close to 850 K. The preferential etching of the *β*-phase can also been explained. Apparently, vanadium is the easiest to be etched and removed, followed by titanium, while aluminum is the most difficult to be etched and removed. The *β*-phase is vanadium rich, compared to the *α*-phase which is aluminum rich. The density of the *α*- and *β*-crystal structures may also play a role in the etch rate. Since the *β*-phase takes on a BCC structure, which is less dense than the HCP structure of the *α*-phase. The increased density means more atoms will be present per unit volume in the *α*-phase. Since the removal mechanism of the plasma etching process is chemical, this increased amount of atom means more reactions will have to take place to remove the same volume of material in the *α*-phase compared to the *β*-phase. The grain size may also have an impact, since the grain boundaries are where the plasma etching process will initiate. Since the *β*-grains are much smaller than the *α*-grains, the percentage of atoms at a grain boundary for the *β*-grains is much higher that of the *α*-grains, meaning layers of material can be removed faster in the *β*-grains.

### Areal plasma etching

**Fig. 9:**
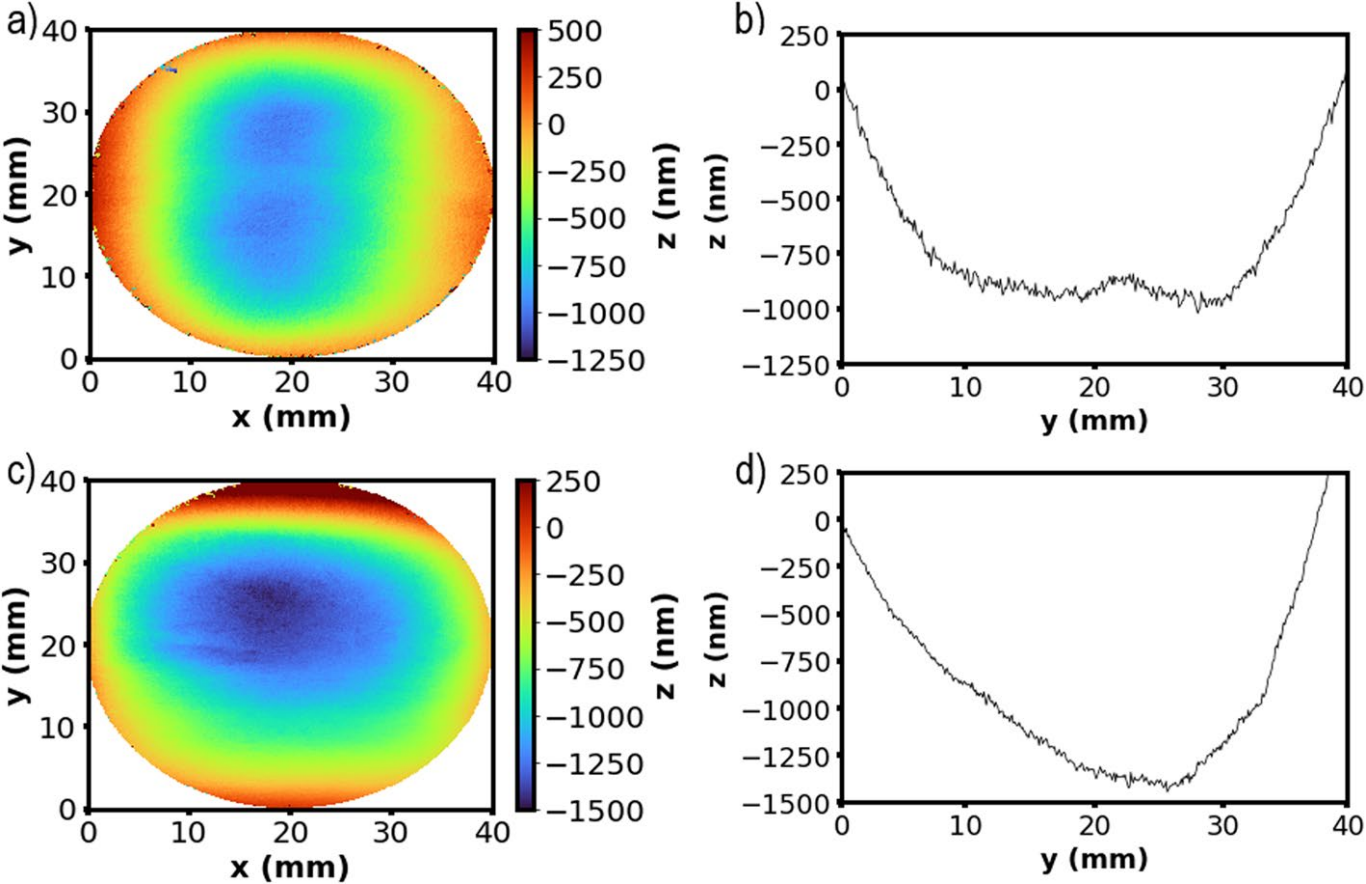
Areal etch using neutral raster paths. **a** Interferometer measurement of plasma etch footprint after three passes, raster at constant speed of 100 mm min^−1^. **b** Cross section at *x* = 20 mm in (**a**). **c** Plasma etch footprint after seven passes, raster at constant speed of 100 mm min^−1^, and (**d**) cross section at *x* = 20 mm in (**c**)

The neutral removal footprints, shown in Fig. 9, display the material removed after (a) three and (c) seven trenches overlapped at 5 mm apart at a beam speed of 100 mm min^−1^. Figure 9b and d displays the cross sections of the footprints at *x* = 20 mm for Fig. 9a and c respectively, showing how etch rate varies with each pass. The trenches started from the bottom right-hand side of the sample and progressing to the top left-hand side. The final pass in Fig. 9c was carried out at *y* = 35 mm which is why the material removal decreases towards the top of the image, and the sharp increase in surface height represents the edge of the Gaussian beam. The three-pass neutral removal footprint (Fig. 9b) shows a relatively flat footprint with ± 100-nm deviation in the material removal over the center 20 mm. This becomes less consistent after the seven-pass raster (Fig. 9c). A distinctive feature in Fig. 9d is the apparent higher etch rate at *y* > 20 mm than *y* < 20 mm. We believe this was due to the higher sample temperatures at *y* > 20 mm than *y* < 20 mm because the raster started at *y* = 5 mm and finished at *y* = 35 mm. To reduce this effect, further etching was carried out using the alternate raster pattern (Fig. S3b).

**Fig. 10:**
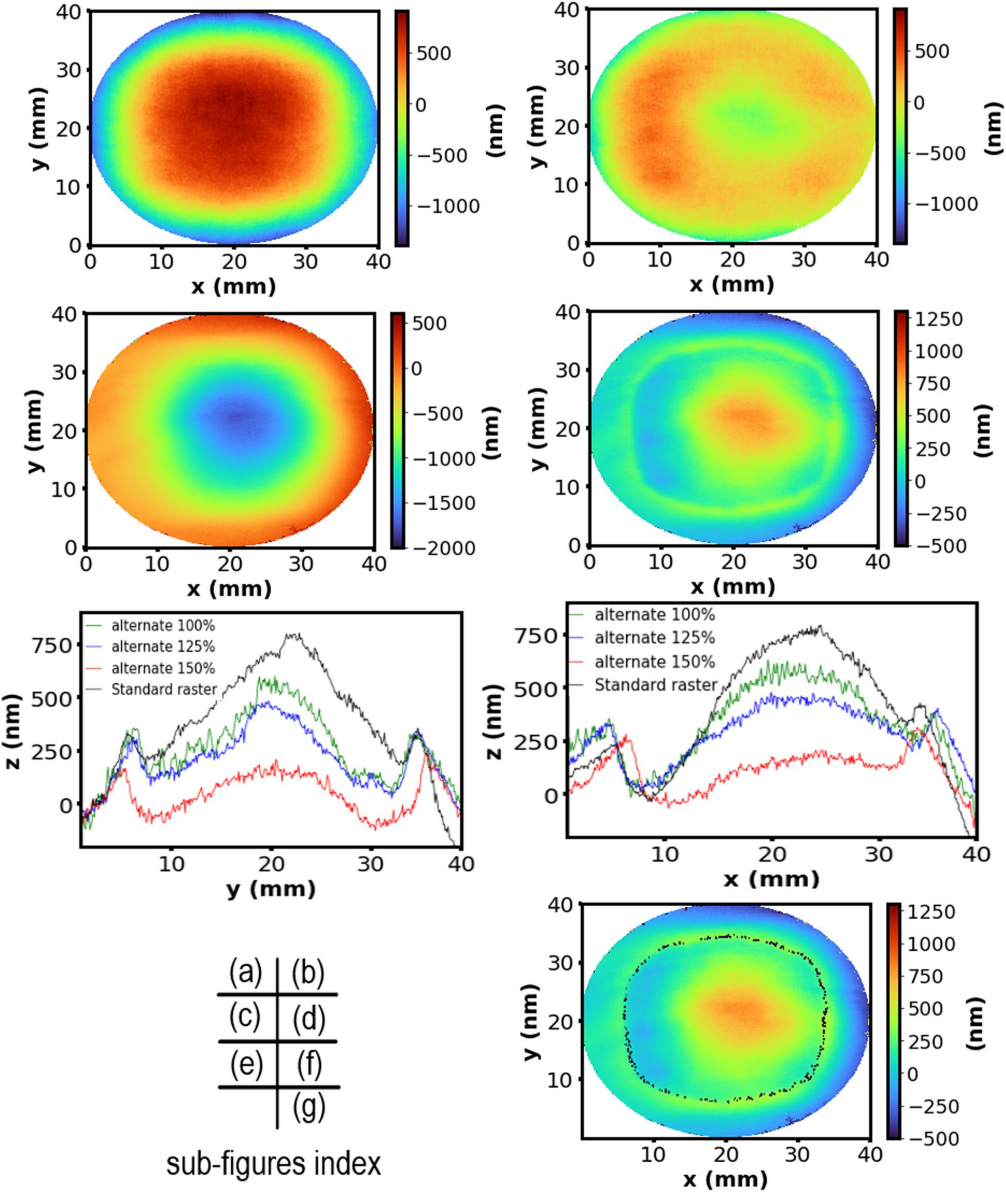
Plasma areal etch experimental and calculated results. Surface measurements of (**a**) unprocessed sample, (**b**) after plasma etching and polishing, (**c**) material removed, and (**d**) the difference between the actual material removed and the material removal as calculated by Eq. 1 after applying the alternate pass raster pattern at the calculated (100%) velocity map. Cross sections of the differences between the actual and the calculated materials removal for 100%, 125%, and 150% of the calculated speeds in (**e**) along *x* and (**f**) *y* directions. The results using the standard raster path at 100% of the calculated speed is also plotted for comparison. (**g**) Illustrating the origin of the ring seen in the etching difference profile (**d**). The points initially measured to be needing 0-nm etching are overlaid to (**d**)

The material removal of the areal plasma etching experiments was compared to a calculated material removal function, predicted using Eq. 1. As discussed in the “ Introduction” section, this equation makes assumptions which are not accurate for the temporal stability, spatial stability, and linearity of the etch rate with respect to time, and it provides a simple estimate which is not computationally intensive and easy to compare the actual and predicted material removals. Figure 10 shows the PSI measurements of the Ti6Al4V surface (a) before plasma etching and (b) after plasma etching and polishing after applying the alternate pass raster pattern at the calculated (100%) velocity map. Figure 10c shows the actual material removed, and Fig. 10d shows the difference between the actual material removal and the calculated (the convolution of the dwell time and the beam function, Eq. 2) material removal (called “the difference” hereafter). In Fig. 10d, the positive values on the *z*-axis correspond to a greater amount of material removed than predicted and vice versa for the negative values. Further experiments were carried out to investigate the effect of processing speed on the “difference” as well as on the flatness of the surface. Figure 10e and f displays the cross-section profiles of the actual and calculated material removal at *x* = 20 mm and *y* = 20 mm for raster speeds of 100%, 125%, and 150% of the calculated processing speed for the alternate raster path. These higher speeds should remove less materials on each scan. The profiles for the raster speed of 100% for the standard neutral removal raster path are also displayed for comparison purpose. A few features can be noted: (i) comparing the samples before and after the plasma etching, the surface form became smoother, or the dome height has been reduced significantly after plasma etch under all the beam speeds; (ii) for 100% and 125% speed, the difference profiles stay in positive for the whole range of 5–35 mm, and only for 150% high speed some parts are below zero; (iii) the shapes for all the three cases are similar, i.e., the difference is the largest at the center of the sample; (iv) the difference between 100 and 125% is smaller than the difference between 125 and 150%; and (v) the profiles are roughly symmetrical for *y* = 20 mm but less so for *x* = 20 mm. The difference when *x* > 20 mm is apparently larger than at *x* < 20 mm; (vi) the difference for the standard raster pattern is larger than the alternate raster pattern.

From the results in Fig. 10, it is apparent that there is significant deviation between the calculated and the actual material removal. The actual removal at the center of the sample is much higher than the calculated and reached a maximum 600 nm at the center (*x* = 20) for the 100% speed using the alternate path. This difference progressively decreases towards the edge of the sample, achieves roughly 0 at a radius about 10 mm from the center, i.e., when *x* = 10 mm, and then it starts to increase again towards the edges of the samples. An “O” ring with a radius about 15 mm centered at the sample center has a local maximum of the difference between the actual and the predicted material removal. We can demonstrate this “O” ring is an artifact due to the mechanical limitation of the motion stage. As the pre-etched surface is dome shaped (due to the edge roll-off effect during hand sanding), the points which require no etching (etching height equals zero) are shaped like an “O” ring, and Fig. 10g displays this “height zero” in black dots overlapped on the image shown in Fig. 10d. The “height zero” positions overlap with the second maximum of the “difference” almost perfectly. This happens at the places where the motion stage is required to rapidly accelerate/decelerate. Theoretically, it requires the stage has an infinitely large speed at those positions so that the etching time is zero, but in practice, the motion stage has a limited speed capability, so it stayed there longer than is required, resulting an etching more than required there.

## Discussion

We believe the above results indicate that the assumption that the beam function can be approximated by a constant Gaussian function is not valid, and temperature dependence needs to be considered. The initially estimated beam moving speed, as calculated from the simple relation height/etch rate, is an overestimate, as this etch rate is obtained from the single trench experiments, and it does not take consideration of the material removal from the adjacent plasma passes in raster. Nor does it take into account the heating contribution when the plasma beam was more than 5 mm (half of the FWHM) away. However, this does not affect the discussion on the difference between the actual and the calculated material removal since the calculated etching using Eq. 1 also considered material removal from the adjacent plasma beams. As the time function is the same for both calculated and actual material removal functions, the calculated material removal should be equal to the actual removal if the idealized 3D Gaussian beam function is the true representation of the practical etching.

A number of reasons can contribute to the fact that the beam function is not a perfect Gaussian function, one being temperature variations. During the plasma etching, the center of the sample becomes hotter than the outer part of the sample due to less heat loss. This leads to an increased rate of chemical reactions at the hotter center according to the Arrhenius equation. Secondly, the dome shape of the pre-etched sample means the calculated speed at the center is the slowest, this means there will be more SF_6_ or fluorine radicals at the center, and this higher concentration of fluorine radicals can also lead to higher chemical reactions there.

Generally speaking, a chemical reaction rate changes with temperature exponentially but linearly with reactant concentration, so we focus our discussion on the sample surface temperature under the plasma beam. According to the above discussion, the difference between the actual and calculated material removal is an indication on the temperature difference between the areal etching and the trench etching (from which the beam function is obtained). If the surface temperature under the plasma beam is higher than the temperature during the trench etching, the difference should be positive. If the temperature is lower than at the trench etching, the difference should be negative. At *y* = 5 mm, during the first pass of the plasma scan for the areal etching, the sample temperature is similar to the trench etching; hence, the difference is close to 0. With subsequent passes, the sample temperature increases due to the heat supplied from the plasma beam; hence, the difference become larger. For the same reason, the difference is negative at the edge of the sample because the temperature is lower than at the trench etching due to the larger heat loss at the edge of the sample. This can also explain why the etching is not symmetrical to *y* = 20 mm for a standard raster pattern. As the raster started from *y* = 5 mm and finished at *y* = 35 mm, the sample temperature should be higher when the plasma beam passed at *y* = 35 mm than at *y* = 5 mm. However, when using the alternate raster pattern, the temperature difference between at the two positions reduces; hence, the etching is much more symmetrical about the *y* = 20-mm axis.

This temperature dependence of the beam function has been investigated for figuring of large optics before. Meister and Arnold (Meister & Arnold 2011) considered both the heat flux from the plasma jet and thermal conduction within the substrate after applying proper boundary conditions and proposed a modified version of Eq. 1, shown in Eq. 5, where p_*x*_(t) and p_*y*_(t) are the current torch position, T(x,y,t) is the spatially and temporally dependent surface temperature field, and B(x,y,T) is the local temperature-dependent beam function (Meister & Arnold 2011). Taking all these factors into account obviously makes the determination of the material removal function far more complex, with reported computational times of 16–20 h.

5 $$R\left(x,y\right)={\int }_{0}^{{t}_{end}}B\left(x-{p}_{x}\left(t\right), y-{p}_{x}\left(t\right), T\left(x,y,t\right)\right)dt$$

From the above discussion, it can be seen that some useful and definite conclusions can be drawn about the beam function using our simple calculation approach. The usually used other approach is to apply a much more calculation demanding de-convolution process to obtain a dwell function using the assumed beam function. The obtained material removal will then be compared to the actual material removal to draw some conclusions about the beam function properties. Another technique is to heat up the sample to an appropriate temperature so that a dynamic equilibrium of the surface temperature is reached quicker under the plasma beam; hence, a stable beam function can possibly be defined (Castelli 2012).

We suggested the formation of the volatile TiF_4_ via the reaction of TiF_3_ with fluorine radicals ^*^F as the main mechanism for the plasma etching of Ti6Al4V alloy. This is a similar mechanism to that of APP etching of silicon-based materials, which has been well established for the manufacturing of semiconductors and optics. Many techniques used for etching silicon-based materials can be adopted for etching of metals such as Ti6Al4V. However, caution needs to be taken when doing so due to significant differences in the thermal conductivity between the silicon-based materials and metals such as Ti6Al4V. The thermal conductivities for all the silicon based optic materials, such as fused silica (1.3 Wm^−1^ K^−1^), quartz (1.3 Wm^−1^ K^−1^), ULE (1.31 Wm^−1^ K^−1^), Zerodur (1.46–1.62 Wm^−1^ K^−1^), and BK7 (1.1 Wm^−1^ K^−1^), are much smaller relative to metals such as Ti, Al, V, and Ti6Al4V which are 15.6, 225, 34.6, and 6.6 Wm^−1^ K^−1^, respectively. This means it will take much longer for a metal sample under the plasma beam to reach a stable temperature or a stable etching rate compared to silicon-based materials. The consequence of this to the beam function nonlinearity requires further study.

## Conclusions

The feasibility of atmospheric pressure plasma etching of Ti6Al4V using an SF_6_ etchant has been demonstrated in this work. The process is shown to be repeatable and leads to significant material removal rates of 0.5 mm^3^ min^−1^–2 mm^3^ min^−1^. A preheating pass using pure argon is required to heat the surface before the etching rate begins to stabilize and for volatile products to be formed. Significant nonlinearity of the etching rate or a nonconstant beam function has been observed during the areal etching, and these are suggested to be related to the temperature dependence of the material removal rate by reactive plasma etching.

The chemistry involved in the process of etching Ti6Al4V surface is complex; however, the key results show preferentially etching of the *β* (BCC)-phase, which is vanadium rich compared to the aluminum-rich (HCP) *α*-phase. This preferential etch is significant, ∼50% greater than the etch rate of the *α*-phase. The plasma etching process also leads to a significant amount of residue being left on the etched surface, comprising of high concentration of oxygen and fluorine at the center of an etched trench. The mechanism for removal of titanium is proposed to be in the form of TiF_4_ via the interaction of fluorine radicals in the plasma jet with the formed TiF_3_.

## Supplementary Information

Additional file 1: Supplementary figures: Figure S1 The atmospheric pressure plasma torch used in this work. a The cross section of the torch; b A image of the torch in operation. Figure S2 Negligible material removal from pure Argon plasma etching. a Footprint and b Profile after 50 mm min^-1^ pure argon plasma trench etch. The torch was moving from right to left at y = 20 mm. Figure S3 Two raster patterns. a The standard and b Alternate pass raster patterns used for areal etching. Figure S4 Optical images of the Ti-6Al-4V samples. a After APP plasma etching; b After the plasma etching and a few seconds hand polishing. Figure S5 A preheating at 50 mm min−^1^ is needed to obtain a clean trench etch. a Trench at 100 mm min−^1^ without any preheating; b Trench at 100 mm min−^1^ with a preheating by pure argon plasma (in both cases the processing direction is right to left). Figure S6 Trench footprint as measured by phase shifting interferometer technique for different beam moving speeds. a 250 mm min^-1^; b 200 mm min^-1^; c 150 mm min^-1^; and d 50 mm min^-1^. Figure S7 SEM images after plasma etching and polishing. The image threshold has been adjusted to show a Only the α phase, b Only the β phase and c Original SEM image from position B in Fig. 5a. Supplementary tables: Table S1. Summary of operational parameters for the plasma torch used in the experiments. Table S2: Summary of the input quantities used for the Factsage simulation. Amount of metals taken as mass of each component in 50 mm x 50 mm x 0.1 mm of Ti6Al4V. Amount of SF_6_ taken as mass per second at a flow rate of 0.8 L per min of Ar(90%)/SF_6_(10%).

## Data Availability

The data that support the findings of this study are available from the first author, upon reasonable request.
