# Supplementary material for: Atmospheric pressure plasma etching of Ti-6Al-4 V using SF6 etchant
Source: J Mater Sci Mater Eng. 2025 Sep 11;20(1):110. doi: 10.1186/s40712-024-00200-9 (PMC12425847; doi:10.1186/s40712-024-00200-9)
Supplement: Supplementary file 1 — Additional file 1: Supplementary figures: Figure S1 The atmospheric pressure plasma torch used in this work. a The cross section of the torch; b A image of the torch in operation. Figure S2 Negligible material removal from pure Argon plasma etching. a Footprint and b Profile after 50 mm min-1 pure argon plasma trench etch. The torch was moving from right to left at y = 20 mm. Figure S3 Two raster patterns. a The standard and b Alternate pass raster patterns used for areal etching. Figure S4 Optical images of the Ti-6Al-4V samples. a After APP plasma etching; b After the plasma etching and a few seconds hand polishing. Figure S5 A preheating at 50 mm min−1 is needed to obtain a clean trench etch. a Trench at 100 mm min−1 without any preheating; b Trench at 100 mm min−1 with a preheating by pure argon plasma (in both cases the processing direction is right to left). Figure S6 Trench footprint as measured by phase shifting interferometer technique for different beam moving speeds. a 250 mm min-1; b 200 mm min-1; c 150 mm min-1; and d 50 mm min-1. Figure S7 SEM images after plasma etching and polishing. The image threshold has been adjusted to show a Only the α phase, b Only the β phase and c Original SEM image from position B in Fig. 5a. Supplementary tables: Table S1. Summary of operational parameters for the plasma torch used in the experiments. Table S2: Summary of the input quantities used for the Factsage simulation. Amount of metals taken as mass of each component in 50 mm x 50 mm x 0.1 mm of Ti6Al4V. Amount of SF6 taken as mass per second at a flow rate of 0.8 L per min of Ar(90%)/SF6(10%). [file 40712_2024_200_MOESM1_ESM.docx]

Atmospheric Pressure Plasma Etching of Ti-6Al-4V

Using SF_6_ Etchant

Alex Bishop ^1^, Zhaorong Huang^1*^, Claudiu Giusca^1^, Adam Bennett ^1^, Marco Castelli^2^, Tian Long See^3^

*^1^ School of Areospace Transport and Manufacturing, Cranfield University, Bedfordshire, MK43 0AL UK.*

*^2^ Centre of Micro/Nano Manufacturing Technology, University College Dublin, Belfield, Dublin 4, Ireland.*

*^3^ The Manufacturing Technology Centre Ltd, Coventry, CV7 9JU, UK.
∗ Correspondence: z.huang@cranfield.ac.uk*


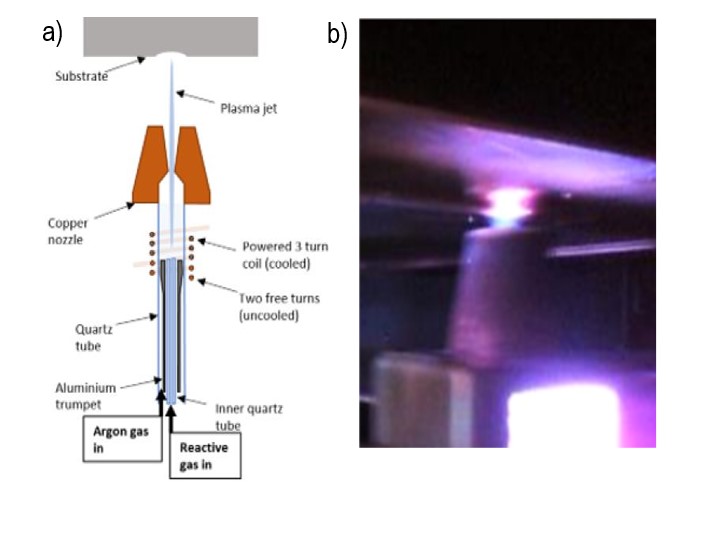


Figure S1 The atmospheric pressure plasma torch used in this work. **a** The cross section of the torch; **b** A image of the torch in operation.

Table S1. Summary of operational parameters for the plasma torch used in the experiments.

| Frequency (MHz) | Power (W) | Argon flow (L min^-1^) | Ar(90%)/SF_6_(10%) (L min^-1^) | work distance (mm) |
| --- | --- | --- | --- | --- |
| 40.25 | 1200 | 20 | 0.8 | 6 |


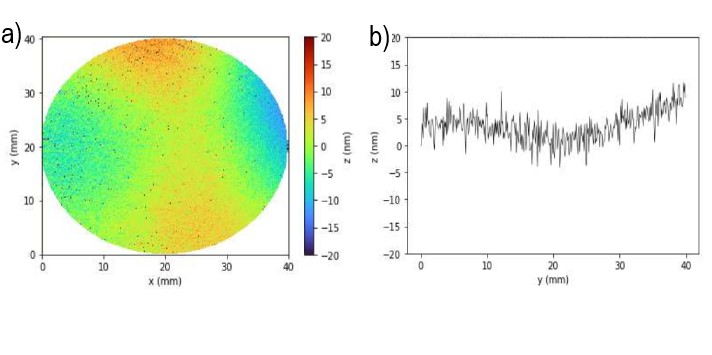


Figure S2 Negligible material removal from pure Argon plasma etching. **a** Footprint and **b** Profile after 50 mm min^-1^ pure argon plasma trench etch. The torch was moving from right to left at y = 20 mm.


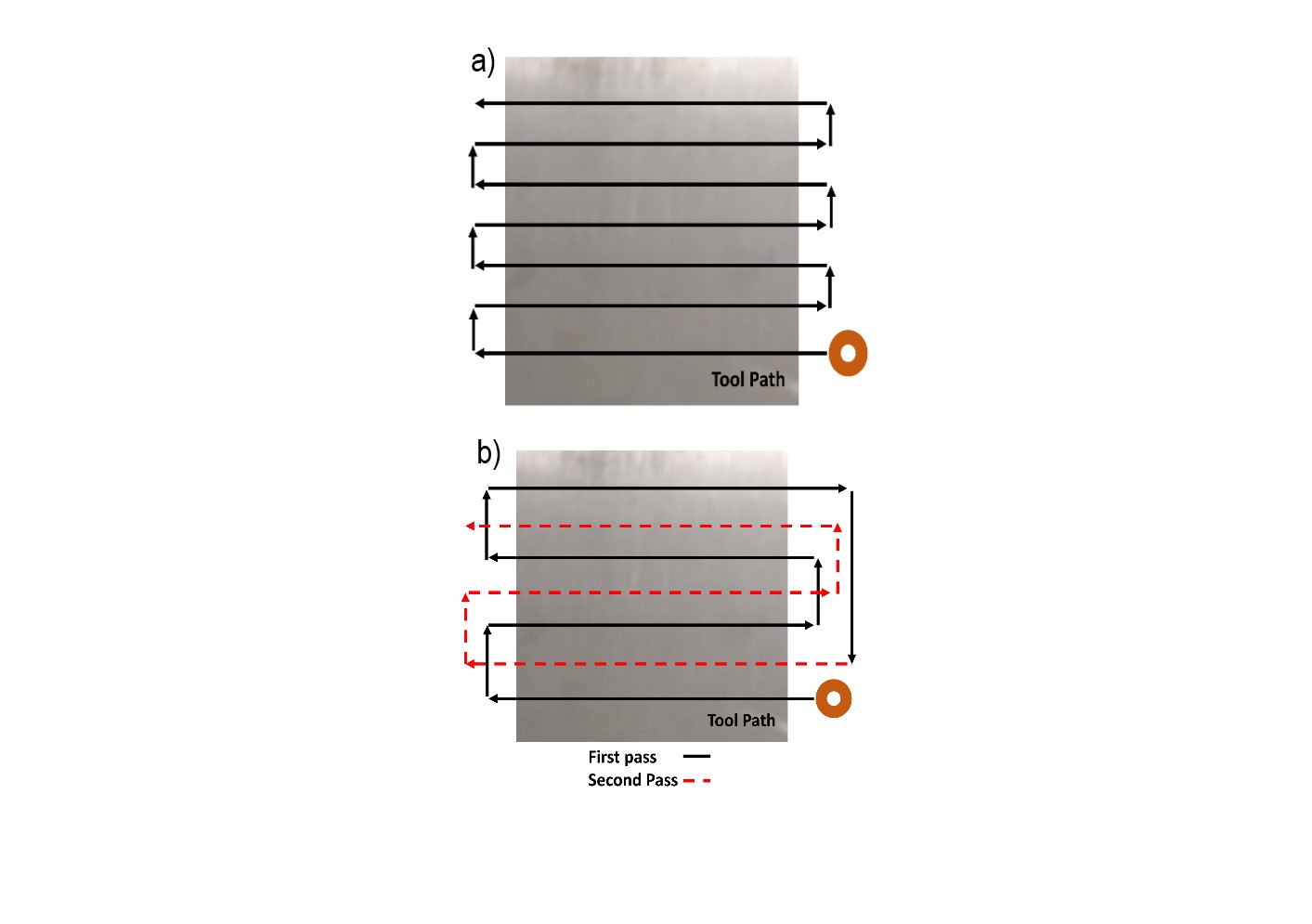


Figure S3 Two raster patterns. **a** The standard and **b** Alternate pass raster patterns used for areal etching.

Table S2: Summary of the input quantities used for the Factsage simulation. Amount of metals taken as mass of each component in 50 mm x 50 mm x 0.1 mm of Ti6Al4V. Amount of SF_6_ taken as mass per second at a flow rate of 0.8 L per min of Ar(90%)/SF_6_(10%).

| Ti (g) | Al (g) | V (g) | SF_6_ (g) |
| --- | --- | --- | --- |
| 1.0373 | 0.0692 | 0.0461 | 0.00855 |


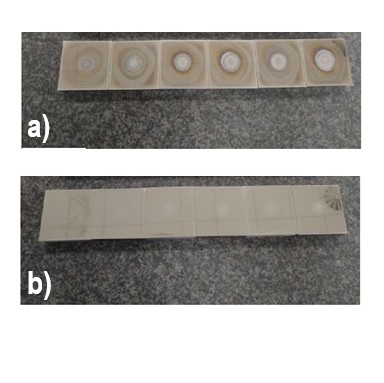


Figure S4 Optical images of the Ti-6Al-4V samples. **a** After APP plasma etching; **b** After the plasma etching and a few seconds hand polishing.


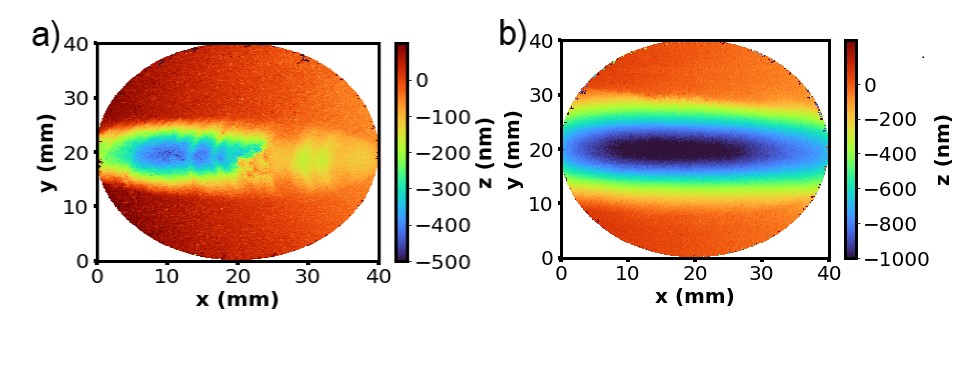


Figure S5 A preheating at 50 mm min−^1^ is needed to obtain a clean trench etch. **a** Trench at 100 mm min−^1^ without any preheating; **b** Trench at 100 mm min−^1^ with a preheating by pure argon plasma (in both cases the processing direction is right to left).


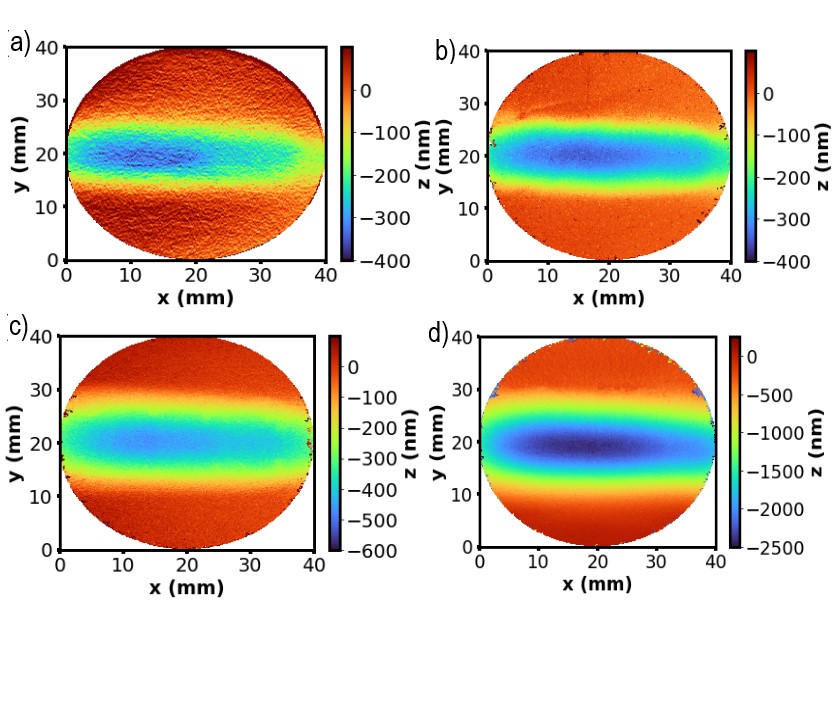


Figure S6 Trench footprint as measured by phase shifting interferometer technique for different beam moving speeds. **a** 250 mm min^-1^; **b** 200 mm min^-1^; **c** 150 mm min^-1^; and **d** 50 mm min^-1^.

Material removal rates (MRR) and the full width at half maximum (FWHM) of a particular plasma etching was obtained by subtracting the surface measurement after plasma treatment and polishing from before any plasma treatment. Each result was the average of 5 repeated measurements to reduce the noise.

Here, σ is the standard deviation across the footprint profile. In Eq. S2, τ_dwell_ is the dwell time, D_max_ is the maximum etch depth, and σ_x_ and σ_y_ are the standard deviation in the x and y directions respectively. In Eq. S3, L is the length of the trench, τ_trench_ is the total time taken to etch the trench, and σ_y_ is the standard deviation of the etched profile across the trench (perpendicular to the direction of etching).


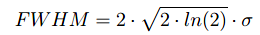
 (Eq. S1)


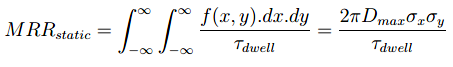
 (Eq. S2)


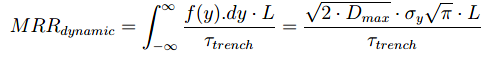
 (Eq. S3)


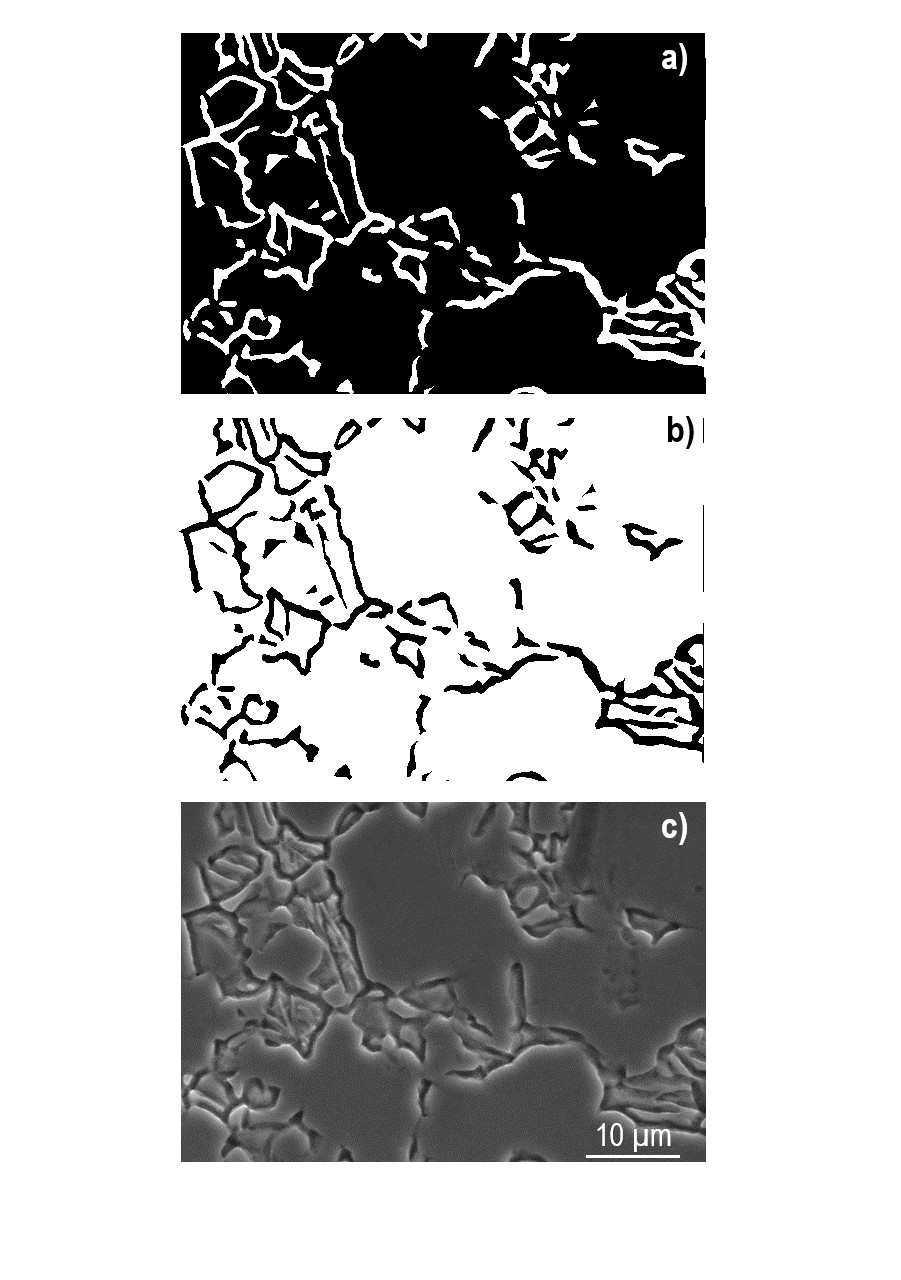


Figure S7 SEM images after plasma etching and polishing. The image threshold has been adjusted to show **a** Only the α phase, **b** Only the β phase and **c** Original SEM image from position B in Fig. 5a.
